# Different nucleos(t)ide analogs in resected hepatitis B virus-associated hepatocellular carcinoma: a systematic review

**DOI:** 10.3389/fphar.2025.1647888

**Published:** 2025-11-14

**Authors:** Hongquan Qiu, Yu Zhang, Fengxia Xu, Songhui Xue

**Affiliations:** 1 Department of Nursing, Nantong Health College of Jiangsu Province, Nantong, Jiangsu, China; 2 Department of Laboratory Medicine, Haimen Hospital Affiliated to Xinglin College of Nantong University, Nantong, Jiangsu, China; 3 Department of General Surgery, The Sixth People’s Hospital of Nantong, Nantong, Jiangsu, China

**Keywords:** hepatitis B virus, hepatocellular carcinoma, antiviral therapy, nucleos(t)ideanalogs, recurrence-free survival

## Abstract

**Background:**

To evaluate the effects of different types of nucleos(t)ide analogs on the survival rate of patients with hepatitis B virus-associated hepatocellular carcinoma (HBV-HCC) after radical resection through a network meta-analysis.

**Methods:**

PubMed, Embase, the Cochrane Library, and CNKI databases were searched up to 6 March 2024. The NOS was used to assess the risk of bias in cohort studies, while the ROB tool in Review Manager was employed for randomized controlled trials. Data on overall survival (OS) and recurrence-free survival (RFS) were extracted from the literature to pool hazard ratios (HRs) and corresponding 95% CrIs. Meta-analysis was performed via R.

**Results:**

24 studies involving 9,787 HBV-HCC patients were included. Compared with the control group, antiviral therapies using telbivudine (HR [95% CrI] = 0.23 [0.12,0.44]), tenofovir disoproxil fumarate (HR [95% CrI] = 0.40 [0.30,0.52]), lamivudine (HR [95% CrI] = 0.50 [0.34, 0.75]), adefovir (HR [95% CrI] = 0.55 [0.38,0.79]), and entecavir (HR [95% CrI] = 0.55 [0.43,0.71]) significantly improved OS. Among these, telbivudine (98.22%) and tenofovir disoproxil fumarate (76.12%) demonstrated superior effects in improving OS. Compared with the control group, antiviral therapies using telbivudine (HR [95% CrI] = 0.45 [0.28,0.70]), tenofovir disoproxil fumarate (HR [95% CrI] = 0.52 [0.44,0.62]), entecavir (HR [95% CrI] = 0.65 [0.55,0.77]),adefovir (HR [95% CrI] = 0.79 [0.65,0.94]),and lamivudine (HR [95% CrI] = 0.82 [0.71, 0.94]) significantly improved RFS. Telbivudine (SUCRA, 93.22%) and tenofovir disoproxil fumarate (SUCRA, 85.37%) exhibited superior effects in improving RFS.

**Conclusion:**

When compared to other nucleos(t)ide analogs, telbivudine and tenofovir disoproxil fumarate exhibited the most notable effects.

**Systematic Review:**

Identifier CRD42024612794.

## 1 Introduction

In Asia, hepatocellular carcinoma (HCC) is most frequently caused by hepatitis B virus (HBV) infection. Treatments such as radical resection, transarterial chemoembolization (TACE), and radiofrequency ablation (RFA) can effectively improve the prognosis of patients with hepatitis B virus-associated hepatocellular carcinoma (HBV-HCC). Nevertheless, despite treatment, the 5-year survival rate remains at only 50%, with a recurrence rate surpassing 70% (Shen et al., 2018). Research has demonstrated that postoperative antiviral therapy using nucleos(t)ide analogs such as lamivudine (LAM), entecavir (ENT), adefovir (ADV), telbivudine (Ldt), and tenofovir disoproxil fumarate (TDF) can suppress the replication of hepatitis B virus deoxyribonucleic acid (HBV-DNA), reduce the HBV-DNA level in the body, improve overall survival (OS) and recurrence-free survival (RFS) in HBV-HCC patients (Choi et al., 2021; He et al., 2019; Huang et al., 2013; Kao et al., 2023; Li et al., 2023; Linye et al., 2023; Qi et al., 2021; Qi et al., 2020; Ren et al., 2018; Rui et al., 2017; Shen et al., 2022; Wang et al., 2022; Xiao et al., 2021; Xu et al., 2019; Zhong et al., 2016; Chen, 2015; Cheng et al., 2011; Ding et al., 2014; Fang et al., 2012; Lin et al., 2016; Zhang, 2015; Huang et al., 2015; Ke et al., 2013; Zhang et al., 2014; Chen et al., 2017), and thereby improve patient prognosis. However, no consensus has been reached on which specific antiviral drug offers the optimal effect for improving the prognosis. Some studies comparing tenofovir and ENT have suggested that TDF is superior to ENT in improving the survival rate and reducing the recurrence rate of HBV-HCC patients (Hu et al., 2022). However, research has reported no significant differences in OS or recurrence rate between the two drugs (Li et al., 2023). Similarly, studies comparing the efficacy of ENT and LAM have reported conflicting results. Research has found similar effects of ENT and LAM on OS in HBV-HCC patients (Shin et al., 2012), while ENT has been reported in other research to provide superior effects for improving OS compared to LAM (Kim et al., 2016). Therefore, we utilized a systematic review and network meta-analysis to integrate existing evidence, aiming to identify the optimal antiviral treatment for affected patients.

## 2 Methods

### 2.1 Data and methods

The present study was carried out as per the Preferred Reporting Items for Systematic Reviews and Meta-Analyses (PRISMA) statement (Liberati et al., 2009), and was pre-registered with the International Prospective Register of Systematic Reviews (PROSPERO) (CRD42024612794).

### 2.2 Literature retrieval

Literature searches were conducted across PubMed, Embase, the Cochrane Library, and CNKI databases, with the search cut-off date on 6 December 2024. The search strategy was based on MeSH and free-text terms, with no language restrictions. The primary search terms included but were not limited to: Hepatitis B virus, hepatocellular carcinoma, overall survival, recurrence-free survival, disease-free survival, progression-free survival, and their corresponding free-text terms.

### 2.3 Literature screening

The articles were first independently evaluated by two researchers, and a third researcher was responsible for consolidating the findings. Studies were included if they met the following criteria (Shen et al., 2018): they were randomized controlled trials (RCTs) or cohort studies (Choi et al., 2021); they involved patients with HBV-HCC who received radical resection as part of their treatment (He et al., 2019); they provided relatively complete follow-up data on OS or RFS (Huang et al., 2013); they used postoperative nucleos(t)ide analog antiviral therapy as the control. Exclusion criteria were as follows (Shen et al., 2018): Patients infected with other types of hepatitis viruses other than hepatitis B virus (Choi et al., 2021); Patients received preoperative antiviral therapy (He et al., 2019); Different patients were treated with different types of antiviral drugs after surgery and were not grouped according to the type; (Huang et al., 2013); Studies that failed to specify the type of antiviral drugs to be used (Kao et al., 2023); The patients received liver transplantation and interferon antiviral therapy (Li et al., 2023); The patients did not receive radical resection. Ultimately, 24 eligible articles were included.

### 2.4 Risk of bias assessment

The selected studies were independently assessed for risk of bias by two researchers, and the results were consolidated by a third researcher. All RCTs were evaluated using the Risk of Bias (ROB) tool in Review Manager, while cohort studies were assessed using the Newcastle-Ottawa Scale (NOS) (Lo et al., 2014). The ROB tool included the following evaluation criteria (Shen et al., 2018): method of random sequence generation (Choi et al., 2021); allocation concealment (He et al., 2019); blinding of participants (Huang et al., 2013); blinding of outcome assessors (Kao et al., 2023); completeness of outcome data (Li et al., 2023); selective outcome reporting (Linye et al., 2023); other potential sources of bias. The NOS included the following evaluation criteria (Shen et al., 2018): representativeness of the exposed group (Choi et al., 2021); representativeness of the non-exposed group (He et al., 2019); ascertainment of exposure (Huang et al., 2013); demonstration that the outcome of interest was not present at the start of the study (Kao et al., 2023); comparability of exposed and non-exposed groups in study design and statistical analysis (Li et al., 2023); assessment of outcome (Linye et al., 2023); adequacy of follow-up duration (Qi et al., 2021); completeness of follow-up in both exposed and non-exposed groups. The results of the risk of bias assessment are presented in Table 1.

**TABLE 1:** Basic characteristics of included studies.

Author	Year	Region	Intervention	Age (mean ± SD)	Gender (♂/♀)	HBV DNA (log copies/mL)	HBeAg (+/−)	Tumor stage	Tumor size (cm)	Child–pugh	Study design	QAT	Outcomes
Ke	2013	China	Lamivudine	48.94 ± 10.47	129/12	4.97	15/126	107/23/11 (BCLC [A/B/C])	4.5	141/0/0 (A/B/C)	Retro	8	OS, RFS
			Control	49.70 ± 12.10	127/14	4.78	16/125	105/26/10 (BCLC [A/B/C])	5	28/14/1 (A/B/C)			
Zhang	2014	China	Entecavir	NA	26/14	NA	NA	NA	4.6	33/6/1 (A/B/C)	Retro	9	OS, DFS
			Control		31/16				4.8	37/10/0 (A/B/C)			
Huang	2015	China	Adefovir	50.6 ± 7.8	90/10	NA	51/49	8/60/32 (BCLC [0/A/B])	4.9	100/0/0 (A/B/C)	RCT	low risk: 2 unclear risk: 2 high risk: 3	OS, RFS
			Control	50.5 ± 8.5	89/11		50/50	8/59/33 (BCLC [0/A/B])	5.1	100/0/0 (A/B/C)			
Ding	2014	China	Entecavir	47.22 ± 10.80	56/18	5.21	NA	NA	NA	54/20/0 (A/B/C)	Retro	6	OS, RFS
			Control	46.49 ± 11.02	30/9	5.02				31/8/0 (A/B/C)			
Lin	2016	China	Entecavir	54.2 ± 2.5	26/9	7.48	NA	NA	NA	28/7/0 (A/B/C)	Retro	9	RFS
			Control	53.1 ± 1.9	20/5	6.94				20/5/0 (A/B/C)			
Fang	2012	China	Lamivudine	48.9 ± 7.41	20/6	6.38	NA	NA	NA	NA	Retro	6	RFS
			Control	50.0 ± 9.63	23/7	6.35							
Cheng	2011	China	Lamivudine	45.32 ± 9.14	38/12	5.56 (5.53–5.64)	NA	NA	NA	NA	Retro	7	RFS
			Control	42.21 ± 8.76	32/11	5.68 (5.50–5.83)							
Chen	2015	China	Lamivudine	52.16 ± 5.14	39/6	NA	NA	38/5/2/0 (AJCC)	NA	41/4/0 (A/B/C)	Pro	7	RFS
			Control	51.91 ± 4.82	36/4			34/5/1/0 (AJCC)		38/2/0 (A/B/C)			
Zhang	2015	China	Lamivudine	52.16 ± 5.14	39/6	NA	NA	38/5/2/0 (TNM)	NA	41/4/0 (A/B/C)	Pro	7	RFS
			Control	51.91 ± 4.82	36/4			34/5/1/0 (TNM)		38/3/0 (A/B/C)			
Xu	2019	China	Entecavir	NA	32/5	NA	NA	24/13 (BCLC [0, A/B, C])	NA	NA	Retro	9	OS, RFS
			Control		31/6			28/9 (BCLC [0, A/B, C])					
Xiao	2020	China	Entecavir	54.34 ± 9.71	51/15	NA	31/35	NA	2.5 ± 0.6	38/17/11 (A/B/C)	Retro	9	OS, PFS
			Control	53.08 ± 9.22	47/19		27/39		2.3 ± 0.7	34/24/8 (A/B/C)			
Ren	2018	China	Entecavir	NA	36/5	NA	NA	40/1 (BCLC [A/B])	NA	NA	Retro	5	OS
			Control		67/8			73/2 (BCLC [A/B])					
Huang	2013	China	Entecavir	50.87 ± 10.07	758/107	NA	512/353	NA	5.21 ± 2.16	NA	Retro	7	DFS
			Adefovir										
			Lamivudine										
			Control	51.75 ± 10.71	156/19		92/83		5.29 ± 1.88				
Qi	2013	China	Entecavir	53.6 ± 5.1	106/45	4.8 ± 1.3	106/45	16/135 (BCLC [0/A])	2.6 ± 0.4	103/48 (A/B)	Pro	8	OS, RFS
			Control	54.2 ± 6.7	61/21	5.0 ± 1.1	61/21	9/73 (BCLC [0/A])	2.6 ± 0.4	53/29 (A/B)			
Rui	2017	China	Lamivudine	51.3 ± 12.0	84/34	NA	NA	NA	7.8 ± 2.3	91/27 (A/B)	Retro	6	OS, TFS
			Control	49.1 ± 12.4	59/25				7.8 ± 2.4	69/15 (A/B)			
Choi	2020	Korea	Tenofovir	54.6 ± 8.6	433/134	2.3 ± 2.4	149/418	142/425 (BCLC [0/A])	2.8	NA	Retro	9	OS, RFS
			Entecavir	54.7 ± 9.3	430/137	2.2 ± 2.3	137/430	151/416 (BCLC [0/A])	2.7				
He	2019	China	Telbivudine	47.74 ± 1.393	67/10	NA	55/22	14/63 (BCLC [0/A])	NA	NA	Retro	7	OS, DFS
			Adefovir	50.87 ± 1.024	90/21		97/14	18/93 (BCLC [0/A])					
He	2023	China	Tenofovir	50.97 ± 12.17	63/11	NA	16/58	32/42 (BCLC [0/A])	2.91 ± 0.82	NA	RCT	Low risk: 2 Unclear risk: 2 High risk: 3	OS, RFS
			Entecavir	49.78 ± 11.95	66/8		22/52	21/53 (BCLC [0/A])	3.05 ± 0.89				
Kao	2022	China	Tenofovir	56.87 ± 8.89	369/63	NA	NA	61/258/113 (BCLC [0/A/B])	NA	423/9 (A/B + C)	Retro	6	OS, RFS
			Entecavir	56.87 ± 5.18	1,162/203			193/814/358 (BCLC [0/A/B])		1,336/29 (A/B + C)			
Li	2023	China	Tenofovir	58.4 ± 10.5	851/138	3.3 ± 1.8	261/728	120/743/126 (BCLC [0/A/B])	4.1 (0.5–23.1)	NA	Retro	8	OS, RFS
			Entecavir	58.3 ± 9.8	844/145	3.3 ± 1.8	270/719	98/747/144 (BCLC [0/A/B])	4.2 (0.3–25.0)				
Qi	2021	China	Tenofovir	49.9 ± 10.7	122/22	6 ± 6.67	29/115	10/107/8/19 (BCLC [0/A/B/C])	5.6 ± 3.8	NA	Retro	9	OS, RFS
			Entecavir	49.3 ± 10.6	247/41	6 ± 6.57	56/232	18/212/19/39 (BCLC [0/A/B/C])	5.4 ± 3.3				
Shen	2022	China	Tenofovir	NA	52/10	NA	15/47	NA	NA	NA	Retro	7	RFS
			Entecavir		450/83		115/418						
Wang	2022	China	Tenofovir	NA	231/34	NA	77/188	22/214/29 (BCLC [0/A/B])	5.5 (0.9–19.5)	NA	Retro	8	OS, RFS
			Entecavir		344/59		117/286	29/330/44 (BCLC [0/A/B])	5.5 (0.8–19.0)				
Zhong	2016	China	Adefovir	49.8 ± 9.9	36/2	4.7 (2.7–5.7)	3/35	NA	4.0 (2.0–10.2)	NA	Retro	7	OS, RFS
			Lamivudine	49 ± 9.7	62/6	5.2 (2.7–6.0)	7/61		4.2 (0.8–12.6)				

Abbreviation: SD, standard deviation; HBV DNA, hepatitis-B virus deoxyribonucleic acid; HBeAg, Hepatitis B e Antigen; cm, centimeters; QAT, quality assessment tools score; OS, overall survival; DFS, disease-free survival; RFS, recurrence-free survival; NA, not available.

### 2.5 Data extraction

Using a pre-designed data collection sheet, data extraction was completed independently by two researchers. Any discrepancies in the extracted data were resolved by consulting a third researcher to ensure consistency. The main extracted data included (Shen et al., 2018): General study information: first author, publication year, and study region (Choi et al., 2021); Basic participant information: sample size, gender composition, age, HBV DNA level, hepatitis B e antigen (HBeAg) status, Child-Pugh score (a clinical grading system used to assess liver function based on parameters such as bilirubin, albumin, prothrombin time, ascites, and hepatic encephalopathy), and antiviral therapies (He et al., 2019); Tumor characteristics: tumor size and stage (Huang et al., 2013); Outcome measures: OS, RFS, or progression-free survival (PFS).

### 2.6 Statistical analysis

In this study, R language was used as the primary data analysis tool, and the “gemtc” package was applied to perform Bayesian network meta-analysis to systematically synthesize and compare the effectiveness of multiple treatment regimens. Model convergence quality was first assessed by calculating the potential scale reduction factor (PSRF). A PSRF value close to 1 indicated that the simulation process had achieved good convergence, ensuring the stability and reliability of the statistical inference results. Then, the ranking of treatment regimens included in the studies was visualized using two methods: first, by calculating and reporting surface under the cumulative ranking curve (SUCRA) values to quantify the overall ranking of each antiviral drug; and second, by utilizing relative effect forest plots and league tables to compare the relative effectiveness of different types of antiviral drugs. Additionally, DFS, RFS, and TFS were combined for statistical analysis. Next, if a closed-loop structure was identified in the network diagram, the “mtc.nodesplit” function was used to perform inconsistency testing. This test was to examine significant differences between indirect and direct evidence to evaluate whether the consistency assumption of the network meta-analysis was valid. At last, the “mtc.anohe” function was applied to perform heterogeneity testing, assessing whether the variability among the included studies exceeded the range of random variation. Potential sources of heterogeneity were further explored to interpret the results more comprehensively.

## 3 Results

### 3.1 Basic information of included studies

**FIGURE 1:**
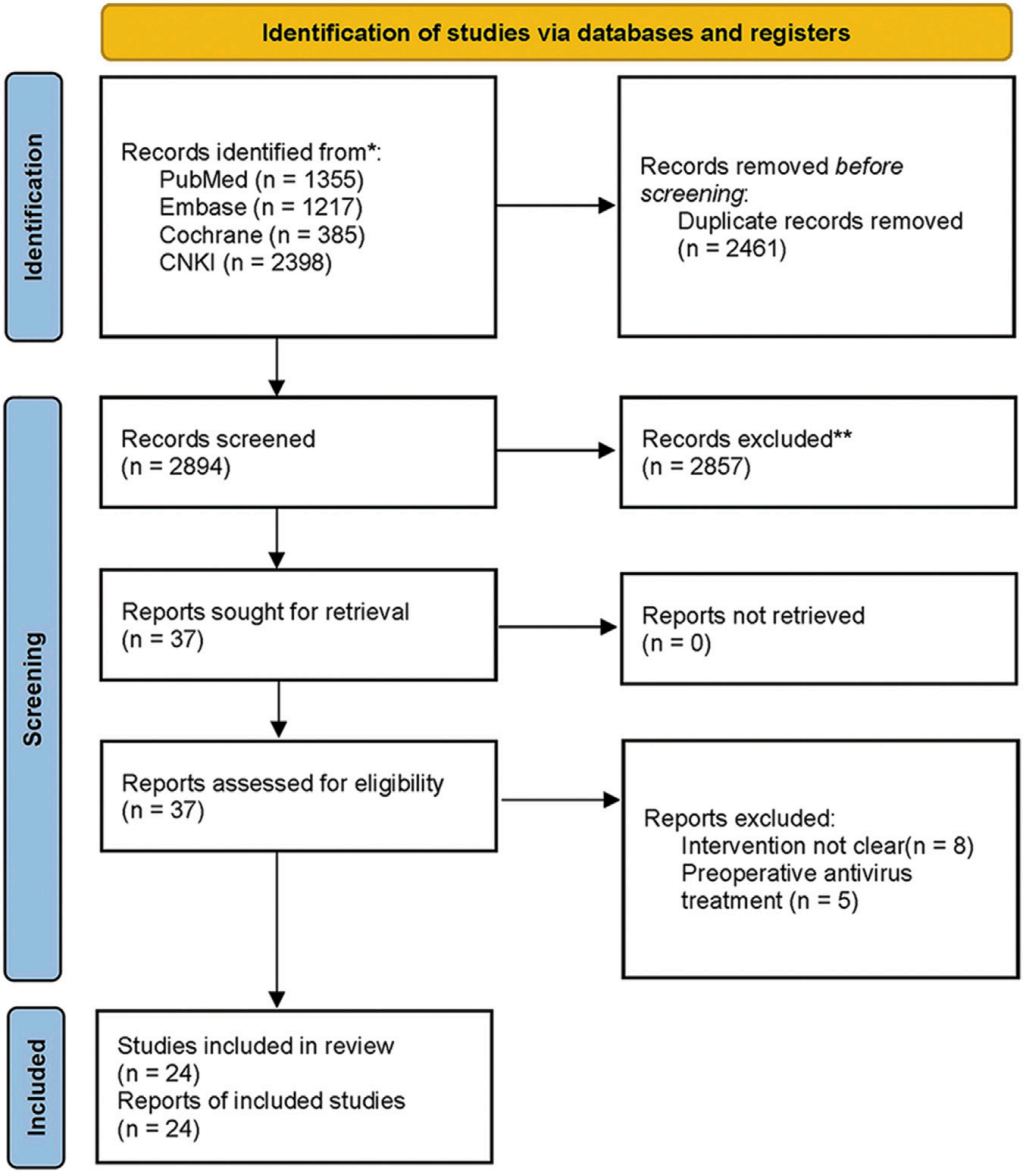
PRISMA 2020 flow diagram.

A total of 5,355 articles were retrieved from the above-mentioned databases. After removing 2,461 duplicates using Endnote 20, 2,894 articles remained. In the first round of screening, 2,857 articles that did not meet the study criteria were excluded after reviewing titles and abstracts, leaving 37 articles. In the second round of screening, after full-text reviews, we excluded 8 articles in which patients had received preoperative antiviral therapy and 5 articles where the types of antiviral drugs were not specified. Ultimately, 24 articles (Choi et al., 2021; He et al., 2019; Huang et al., 2013; Kao et al., 2023; Li et al., 2023; Linye et al., 2023; Qi et al., 2021; Qi et al., 2020; Ren et al., 2018; Rui et al., 2017; Shen et al., 2022; Wang et al., 2022; Xiao et al., 2021; Xu et al., 2019; Zhong et al., 2016; Chen, 2015; Cheng et al., 2011; Ding et al., 2014; Fang et al., 2012; Lin et al., 2016; Zhang, 2015; Huang et al., 2015; Ke et al., 2013; Zhang et al., 2014) meeting the criteria were included in the study. The 24 included studies were published between 2011 and 2023, involving a total of 9,787 patients. The literature retrieval and screening process are illustrated in Figure 1. The included patients were predominantly from China, except for one study conducted in South Korea. The average age of the patients was approximately 50 years, and most were classified as Child-Pugh grade A. The majority of the studies were retrospective. Detailed baseline characteristics are presented in Table 1.

### 3.2 Risk of bias assessment

Of the 22 cohort studies included, 6 studies achieved a score of 9 on the NOS, 4 studies scored 8, 7 studies received a score of 7, 4 studies scored 6, and 1 study obtained a score of 5. Some studies had issues, including incomplete control of confounding factors, lack of descriptions regarding loss to follow-up, or short follow-up durations. The two included RCTs were rated as having a low risk of bias in 2 domains, unclear risk in 2 domains, and high risk in 3 domains. Both RCTs were open-label studies, with a high risk of bias in the domains of blinding and allocation concealment.

### 3.3 Effect of antiviral therapy on OS in HBV-HCC patients

**FIGURE 2:**
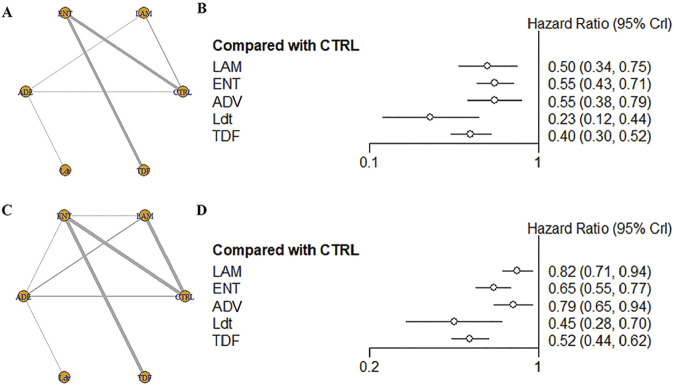
**(A)** Network structure diagram of different antiviral therapies; **(B)** Forest plot of different antiviral therapies; **(C)** Network structure diagram of different antiviral therapies; **(D)** Forest plot of different antiviral therapie.

For OS, a total of 17 studies, including 7,890 patients and five intervention measures, were included in the analysis (Figure 2A). Heterogeneity analysis and inconsistency testing using the node-splitting method demonstrated that the network meta-analysis satisfied the assumptions of homogeneity and consistency. Compared with the control group, antiviral therapies using Ldt (HR [95% CrI] = 0.23 [0.12, 0.44]), TDF (HR [95% CrI] = 0.40 [0.30, 0.52]), LAM (HR [95% CrI] = 0.50 [0.34, 0.75]), ADV (HR [95% CrI] = 0.55 [0.38, 0.79]), and ENT (HR [95% CrI] = 0.55 [0.43, 0.71]) significantly improved OS (Figure 2B; Table 2). The SUCRA-based probability rankings were consistent with the trends observed in the forest plot and league table. Ldt showed the best effect in improving OS (SUCRA = 98.22%).

**TABLE 2:** League table of OS following different antiviral therapies.

	Control	LAM	ENT	ADV	Ldt	TDF
Control						
LAM	1.99 (1.34, 2.97)					
ENT	1.81 (1.41, 2.31)	0.91 (0.57, 1.44)				
ADV	1.82 (1.26, 2.62)	0.91 (0.57, 1.45)	1.01 (0.65, 1.57)			
Ldt	4.34 (2.26, 8.38)	**2.18 (1.07, 4.45)**	**2.4 (1.2, 4.85)**	**2.38 (1.39, 4.12)**		
TDF	2.51 (1.91, 3.3)	1.26 (0.78, 2.03)	**1.39 (1.24, 1.56)**	1.38 (0.87, 2.18)	0.58 (0.28, 1.17)	

Bold indicates that the row intervention is significantly different from the column intervention.

### 3.4 Effect of antiviral therapy on RFS in HBV-HCC patients

For RFS, a total of 23 studies, including 9,671 patients and five intervention measures, were included in the analysis (Figure 2C). Heterogeneity analysis and inconsistency testing using the node-splitting method demonstrated that the network meta-analysis satisfied the assumptions of homogeneity and consistency. Given that most studies reported RFS, we treated DFS and TFS as RFS for analysis. Compared with the control group, antiviral therapies using Ldt (HR [95% CrI] = 0.45 [0.28, 0.70]), TDF (HR [95% CrI] = 0.52 [0.44, 0.62]), ENT (HR [95% CrI] = 0.65 [0.55, 0.77]), ADV (HR [95% CrI] = 0.79 [0.65, 0.94]), and LAM (HR [95% CrI] = 0.82 [0.71, 0.94]) significantly improved RFS (Figure 2D and Supplementary Table S1). The SUCRA-based probability rankings were consistent with the trends observed in the forest plot and league table. Ldt showed the best effect in improving RFS (SUCRA = 93.22%).

## 4 Discussion

The results of this study indicated that nucleos(t)ide analogs, such as LAM, ENT, ADV, Ldt, and TDF, significantly improved OS and RFS in HBV-HCC patients after radical resection, compared to those who did not receive nucleos(t)ide analog antiviral therapy. A key mechanism of HBV-induced carcinogenesis is the extensive replication of HBV in the body following infection. This process leads to the integration of HBV DNA into the host hepatocyte genome, triggering the activation of proto-oncogenes and impairing the function of tumor suppressor genes, thereby driving the development and progression of liver cancer (Jiang et al., 2021). In HBV-HCC patients, after taking nucleos(t)ide analog antiviral drugs, these analogs are converted into triphosphate compounds through catalysis by cellular kinases. Due to the absence of a 3′-hydroxyl group in their ribose molecules, the triphosphate compounds, once integrated into viral DNA, cause the termination of DNA chain elongation. This process inhibits HBV-DNA replication and effectively reduces the HBV viral load (Levrero and Zucman-Rossi, 2016). Studies have found that high HBV viral load in the serum can promote the growth and metastasis of HCC (Su et al., 2013; Huang et al., 2011). Radical resection alters the immune status of HBV-HCC patients, making them prone to HBV reactivation and high HBV viral load replication postoperatively (Li et al., 2020; Wang et al., 2023). Nucleos(t)ide analogs, which can inhibit HBV-DNA replication, effectively reduce the HBV-DNA level, thereby improving OS and RFS in these patients. Additionally, treatment with nucleos(t)ide analogs has been shown to reduce the levels of regulatory T cells (Tregs) in the peripheral blood of patients with chronic hepatitis B (He and Zhao, 2019; Xu, 2021). Research has indicated that CD4^+^CD25^high^ regulatory T cells suppress Th cell responses and HBV-specific cytotoxic T lymphocyte-mediated immune responses, which are considered key contributors to HBV immune tolerance (Cheng et al., 2023; Yu, 2018). Nucleos(t)ide analogs can decrease the levels of peripheral Tregs, thereby reducing immune tolerance, modulating the patient’s immune status (He and Zhao, 2019), and facilitating viral clearance in HBV-HCC patients. This mechanism contributes to the improvement of OS and RFS in these patients.

Furthermore, our study findings suggested that different types of nucleos(t)ide analogs exhibited varying effects on improving OS and RFS in HBV-HCC patients. LAM, ENT, and ADV were less effective than Ldt in improving OS, while ENT was less effective than TDF. In terms of improving RFS, LAM was less effective than ENT, and both LAM and ADV were less effective than Ldt. Moreover, LAM, ENT, and ADV were all less effective than TDF. These differences may be attributed to variations in the specific mechanisms by which different nucleos(t)ide analogs inhibit HBV-DNA replication. LAM is a cytosine analog that inhibits DNA replication by competitively binding to the active site of HBV reverse transcriptase, thereby blocking its activity. ADV is an adenosine monophosphate analog that competes with natural nucleoside triphosphates (NTPs) to inhibit HBV DNA polymerase. ENT is a guanosine analog that competes with dGTP for HBV DNA polymerase. Ldt is a thymidine deoxynucleotide analog that competes with HBV’s natural substrate, thymidine 5′-adenosine. TDF is a diphosphate of fovir, which competes for incorporation into the viral DNA chain. Due to the absence of a 3′-OH group, tenofovir blocks DNA chain elongation and inhibits viral replication (Hruba et al., 2023; Zhang et al., 2019). Since HBV is a retrovirus, its reverse transcriptase lacks proofreading capability and cannot correct mismatched nucleotides (Yasutake et al., 2020). This characteristic leads to the presence of viral strains with diverse genetic sequences in HBV-HCC patients. During long-term postoperative use of nucleos(t)ide analogs, drug-resistant strains with greater survival ability and replication potential are gradually selected. Consequently, drug resistance frequently develops, leading to reduced sensitivity of the virus to antiviral therapy (Song et al., 2012; Liu et al., 2021). Clinical trial evidence has indicated that the rates of drug resistance vary depending on the type of nucleos(t)ide analog used in treatment. During LAM treatment, the drug resistance rate was approximately 20% after 1 year and increased to as high as 70% after 5 years. Furthermore, about 50% of patients who develop LAM resistance would also develop resistance to ENT within 5 years of treatment (Amini-Bavil-Olyaee et al., 2010). For ADV, the resistance rate was around 2% after 2 years and approximately 29% after 5 years (Roediger et al., 2022). During Ldt, the resistance rate was 11% after 2 years (Tacke and Kroy, 2016). For ENT, the resistance rate was 1.2% after 5 years of treatment (Takayama et al., 2021), while TDF showed a similarly low resistance rate during treatment (Li et al., 2021). This may be related to the poorer effectiveness of LAM in improving OS and RFS, compared to the better outcomes observed with Ldt, TDF, and ENT. The differences in resistance rates among various nucleos(t)ide analogs may be attributed to their specific resistance mechanisms and mutation sites. For LAM, resistance is primarily associated with mutations at rtM204I/V in the POL/RT region. ADV resistance is linked to mutations at rtN236T in the D domain. ENT resistance develops after the emergence of the rtM204V + rtL180M mutations. Ldt resistance is associated with mutations at rtL80I and rtL80V, while TDF resistance is related to mutations at rtP177G and rtF249A (Li et al., 2021).

This study has certain limitations. First, most of the included studies are cohort studies, with relatively few RCTs addressing the topic. Second, some of the included studies had small sample sizes. The study did not ascertain the effects of other non-nucleoside antiviral drugs, such as interferons, or combination therapy regimens due to the limited number of studies. All participants were from East Asian populations, limiting the generalizability of the conclusions. Caution is needed when applying these findings to populations in other regions. Future research should consider including multinational data to enhance global applicability. Third, many of the included RCTs were open-label designed, with no mention of allocation concealment or blinding methods, raising the possibility of implementation and measurement biases, which could affect the reliability of the results. Fourth, the outcome measures did not account for the incidence of adverse events, as only a few studies provided relevant data. Fifth, owing to the small number of included studies, publication bias was not examined. Sixth, excluding preoperative antiviral therapy may affect the external validity of the findings, as preoperative treatment is clinically significant in preventing viral reactivation and improving prognosis. Future research could consider evaluating the impact of preoperative treatment.

## 5 Conclusion

Different types of nucleos(t)ide analogs can improve both OS and RFS in HBV-HCC patients after radical resection. Although the findings of this study suggest that Ldt and TDF exhibit the most notable effects on OS and RFS in HBV-HCC patients, it may cause drug resistance. As the Ldt therapy is used continuously, drug resistance may occur, which may limit its long-term effectiveness. Due to the limitations of this study, the results of this study should be interpreted with caution. Clinicians should be cautious when applying these findings to long-term treatment plans. Further research is needed to explore strategies to prevent and manage drug resistance in these patients.

## Data Availability

The original contributions presented in the study are included in the article/Supplementary Material, further inquiries can be directed to the corresponding author.
