# Supplementary material for: Different nucleos(t)ide analogs in resected hepatitis B virus-associated hepatocellular carcinoma: a systematic review
Source: Front Pharmacol. 2025 Nov 14;16:1647888. doi: 10.3389/fphar.2025.1647888 (PMC12660110; doi:10.3389/fphar.2025.1647888)
Supplement: Supplementary file 1 [file Table1.docx]

**Table S1.** League table of RFS following different antiviral therapies

|  | **Control** | **LAM** | **ENT** | **ADV** | **Ldt** | **TDF** |
| --- | --- | --- | --- | --- | --- | --- |
| Control |  |  |  |  |  |  |
| LAM | 1.22 (1.06, 1.41) |  |  |  |  |  |
| ENT | 1.53 (1.3, 1.8) | **1.25 (1.01, 1.55)** |  |  |  |  |
| ADV | 1.27 (1.06, 1.53) | 1.04 (0.83, 1.3) | 0.83 (0.65, 1.07) |  |  |  |
| Ldt | 2.24 (1.42, 3.5) | **1.83 (1.14, 2.92)** | 1.46 (0.9, 2.36) | **1.76 (1.16, 2.66)** |  |  |
| TDF | 1.92 (1.61, 2.29) | **1.57 (1.25, 1.96)** | **1.25 (1.19, 1.33)** | **1.51 (1.17, 1.94)** | 0.86 (0.53, 1.4) |  |
